# New England Salt Marsh Recovery: Opportunistic Colonization of an Invasive Species and Its Non-Consumptive Effects

**DOI:** 10.1371/journal.pone.0073823

**Published:** 2013-08-29

**Authors:** Tyler C. Coverdale, Eric E. Axelman, Caitlin P. Brisson, Eric W. Young, Andrew H. Altieri, Mark D. Bertness

**Affiliations:** 1 Department of Ecology and Evolutionary Biology, Brown University, Providence, Rhode Island, United States of America; 2 Smithsonian Tropical Research Institute, Balboa, Ancon, Republic of Panama; University of British Columbia, Canada

## Abstract

Predator depletion on Cape Cod (USA) has released the herbivorous crab *Sesarma* *reticulatum* from predator control leading to the loss of cordgrass from salt marsh creek banks. After more than three decades of die-off, cordgrass is recovering at heavily damaged sites coincident with the invasion of green crabs ( *Carcinusmaenas* ) into intertidal *Sesarma* burrows. We hypothesized that *Carcinus* is dependent on *Sesarma* burrows for refuge from physical and biotic stress in the salt marsh intertidal and reduces *Sesarma* functional density and herbivory through consumptive and non-consumptive effects, mediated by both visual and olfactory cues. Our results reveal that in the intertidal zone of New England salt marshes, *Carcinus* are burrow dependent, *Carcinus* reduce *Sesarma* functional density and herbivory in die-off areas and *Sesarma* exhibit a generic avoidance response to large, predatory crustaceans. These results support recent suggestions that invasive *Carcinus* are playing a role in the recovery of New England salt marshes and assertions that invasive species can play positive roles outside of their native ranges.

## Introduction

Unchecked human population growth has threatened the persistence of natural ecosystems [1] by escalating extinctions [2], ecosystem phase shifts [3], habitat loss [4], and species invasions [5]. Species introductions can have negative ecological impacts and, consequently, are often viewed as destructive [6,7]. Recently however, invasive species have been shown to restore lost ecological functions and promote recovery within heavily degraded ecosystems [8], stimulating debate on the costs and potential benefits of species outside native ranges e.g. [6,9–12], particularly in light of the extent and severity of human impacts on ecosystems.

Ecosystem recovery after anthropogenic disturbance has been documented in terrestrial [13], freshwater [14] and marine systems [15], but full recovery has been observed in only a third of ecological recovery studies [16]. Invasive predators could have a particularly large impact on the recovery of degraded communities if their impact is exerted through both consumptive and non-consumptive effects [17]. Non-consumptive effects have been hypothesized to be a more potent community structuring force than predation alone because a single predator can influence more prey through non-consumptive interactions than it can consume directly, resulting in larger community effects [17,18]. Elucidating recovery mechanisms, including the potential for invasive species to aid in recovery, is essential for informing conservation to improve management success, attain sustainable human ecosystem use, and test ecological theory [19,20].

Overexploitation of predators is one of the greatest threats to coastal marine ecosystems [21], so the resilience and recovery potential of ecosystems damaged by predator depletion is of considerable conservation and management importance [22,23]. The importance of predators on coral reefs [24] and kelp forests [25] is well established, but their role in salt marshes remains contentious [26,27]. Recent die-offs of salt marsh cordgrass across the western Atlantic [28–30], however, illustrate that in the absence of top predators salt marshes can be heavily damaged by herbivory. Such results suggest that, under continued predator depletion, salt marshes worldwide may become vulnerable to consumer-driven die-off [27].

Herbivore-driven die-offs on Cape Cod (MA), first reported in 2002, result from overgrazing by the native, nocturnal marsh crab *Sesarma* *reticulatum* on the low marsh cordgrass *Spartina* *alterniflora* [30,31], the foundation species critical for New England marsh growth and the provisioning of ecosystem services. *Sesarma* are common in New England, but die-off is not found in undisturbed salt marshes with robust predator populations and low *Sesarma* densities [32–34]. At sites with heavy recreational fishing >50% of marine predators (e.g. striped bass *Morone* *saxatilis* , blue crab *Callinectes* *sapidus* ) have been removed, increasing *Sesarma* densities by ~400% and triggering cordgrass die-off [32]. On Cape Cod, *Sesarma*-driven die-off has denuded >95% of creek banks at impacted sites and is prevalent at >90% of marshes regionally [31]. At elevated densities, *Sesarma* dig communal burrow networks in denuded peat banks. Burrows can displace >65% of peat volume and large burrow complexes can contain >25 *Sesarma*, which rely on this refuge from predation and desiccation to persist in the marsh intertidal [35].

Recently, invasive European green crabs ( *Carcinusmaenas* ) have colonized the intertidal zone of sites with high *Sesarma* densities. Although *Carcinus* do not dig burrows, they have been shown to use *Sesarma* burrows and evict resident crabs. *Carcinus* are >50X more common in the intertidal zones of die-off than healthy sites, where *Sesarma* burrow density is >5X greater [36]. These sites lack robust predator populations, have high *Sesarma* densities and have experienced severe cordgrass die-off over the last ~35 years [32,33]. Recently, sites colonized by *Carcinus* have experienced cordgrass regrowth, suggesting that *Carcinus* may act as compensatory predators [36], restoring predation pressure lost to localized overfishing for recreationally targeted species.

Our previous work suggests that the interaction between *Sesarma* and *Carcinus* is largely dictated by a behavioral response of *Sesarma* to the presence of, but not predation by, *Carcinus* [36]. In this paper we test the hypothesis that *Carcinus* opportunistically utilize *Sesarma* burrows for refuge and non-consumptively reduce *Sesarma* activity and herbivory through olfactory and visual cues. Specifically, we hypothesized that in the intertidal (1), *Carcinus* displace *Sesarma* from their burrows to avoid predation and desiccation, allowing them to remain in the intertidal during low tide (2), *Carcinus* play a compensatory predation role by reducing *Sesarma* functional density and herbivory and (3) non-consumptive interactions between *Sesarma* and *Carcinus* are mediated by olfactory and/or visual cues.

## Methods

### Why do *Carcinus* use *Sesarma* burrows?

To test the hypothesis that *Carcinus* use *Sesarma* burrows as a refuge habitat from desiccation and/or predation in the intertidal, we ran a fully factorial tethering experiment crossing burrow and predator exclusion at two heavily burrowed sites. Predator exclusion cages (40 x 40 x 40 cm) and burrow exclusion panels (40 x 40 cm) were constructed of 12 mm galvanized hardware cloth. Cages and burrow exclusion panels were attached to the marsh surface with garden staples to prevent access by burrowing predators and the escape of tethered *Carcinus*. *Carcinus* were tethered with 15 cm of 50 lb braided fishing line threaded between the second and third walking legs and attached to the carapace with cyanoacrylic glue. Carapace pieces attached to the tether at the end of the experiment provided evidence of predation, while dead intact *Carcinus* were evidence of physical stress-induced mortality. Previous tethering experiments revealed that crab behavior and survivorship are unaffected by this tethering method [30]. Predator exclusion cages prevented predation but allowed access to *Sesarma* burrows, while burrow exclusion panels prevented burrow use by tethered *Carcinus* and allowed access to predators. Burrow densities at both sites were >115/m^2^ and tethered *Carcinus* with access to burrows immediately entered them when deployed. Tethered *Carcinus* were randomly assigned to one of four treatments: open (burrow access and predator exposure), burrow exclusion (hardware cloth floor preventing burrow access), predator exclusion (cage preventing predator access), and predator and burrow exclusion (n=15/treatment/site). *Carcinus* mortality was scored after 48 hours and analyzed with a two-factor ANOVA (caged vs. uncaged and burrow access vs. burrow exclusion).

To examine the generality of *Carcinus* reliance on *Sesarma* burrows, we surveyed creek bank *Carcinus* and *Sesarma* densities at healthy and die-off sites (n=3 sites/site type) in 2011. At each site, three replicate creek banks (10 m long, 1 m wide and 1 m deep) were surveyed for *Carcinus* and *Sesarma*. Species-specific abundances (*Carcinus* or *Sesarma*) were pooled by site. Species-specific abundance was analyzed with a one-factor ANOVA (healthy vs. die-off sites). To examine how *Carcinus* abundance varies temporally, sites were surveyed again in 2012. *Carcinus* abundance per creek bank was aligned rank transformed using ARTool [37] for nonparametric factorial data analysis and analyzed with ANOVA (site-type, year, and site-type*year).

### Does the presence of *Carcinus* reduce *Sesarma* functional density and herbivory?

To test the hypothesis that *Carcinus* reduce *Sesarma* activity and herbivory we performed a *Carcinus* addition experiment at Blackfish Creek (Wellfleet, MA), a die-off site with little recovery and few naturally occurring *Carcinus* (7.3 ± 4.3 crabs/100 m^2^). We randomly selected 20 plots on creek banks with conspicuous *Sesarma* herbivory, separated by >4 meters. Ten plots were randomly assigned as *Carcinus* additions and the others assigned as unmanipulated controls. All plots had high fiddler crab (*Uca pugnax*) densities, so both *Carcinus* addition and control plots had high ambient crab activity. *Carcinus* placed in addition plots were of similar size to the large *Carcinus* used in avoidance response trials and predation experiments described below. To assess how the presence of *Carcinus* affects the spatial extent of *Sesarma* herbivory, we transplanted 3 cores (7.5 cm diameter) of cordgrass into each plot 0, 0.5 and 1.0 m from the center, parallel to the shore. In crab addition plots, a tethered *Carcinus* was added to the center on a 25 cm tether and provided with an artificial burrow for refuge. We checked all replicates biweekly for *Carcinus* survival and signs of predation on *Sesarma*. *Carcinus* were replaced as necessary throughout the experiment to ensure constant presence of live *Carcinus* in addition plots. We quantified *Sesarma* activity by sampling functional *Sesarma* density 0, 0.5 and 1.0 m from each plot’s center with pitfall traps [32]. Functional densities were measured before and 24 hours after *Carcinus* addition to test the hypothesis that the presence of *Carcinus* reduces *Sesarma* activity. After a month, the number of stems grazed by *Sesarma* on each cordgrass culm was quantified to test the hypothesis that the presence of *Carcinus* reduces *Sesarma* herbivory and that this effect decreases with distance from *Carcinus*. *Sesarma* functional density and herbivory were analyzed with 2-factor ANOVAs (treatment x distance).

### What cues trigger an avoidance response by *Sesarma*?

We performed avoidance response trials in field mesocosms to test the hypothesis that non-consumptive effects mediate interactions between *Sesarma* and *Carcinus*. Mesocosms had opaque sides and mimicked the submerged intertidal but were flat and lacked burrows to allow quantification of escape time in the absence of refugia. Trials were performed shortly after dusk because *Sesarma* are nocturnal crabs and leave their burrow complexes at night to forage. Mesocosms were supplied with fresh seawater for each trial to avoid the accumulation of olfactory cues. An arena was established within the mesocosm and its size (17 cm radius) was based on the average distance to the nearest burrow in field plots (9.8±0.5 cm). *Sesarma* (2.0±0.2 cm carapace width) were placed in the center of the mesocosm under a smaller container to allow time for habituation after which the smaller container was removed and the time for each *Sesarma* to move outside the arena was recorded. To examine whether the induction of *Sesarma* avoidance behavior is species-specific, trials were run with three similarly sized large predatory crabs, *Carcinus* (7.0 cm carapace width), Atlantic rock crab (*Cancer irroratus*; 9.6 cm carapace width) and blue crab ( *Callinectes* *sapidus* ; 13.2 cm carapace width), as well as two non-predatory crabs commonly found in New England marshes: the horseshoe crab ( *Limulus* *polyphemus* ; 7.8 cm carapace width), and spider crab ( *Libinia* *emarginata* ; 5.8 cm carapace width). To test whether *Sesarma* avoidance response is size specific, trials were run with small (3.9 cm) and large (7.0 cm) *Carcinus*. To test the mechanism(s) of avoidance behavior, visual and olfactory cues were isolated in separate trials. For visual trials, a large *Carcinus* was placed in a clear, sealed glass container visible to *Sesarma*; for olfactory trials, water with *Carcinus* effluent was released into the mesocosm prior to the insertion of *Sesarma*. Avoidance responses were compared against control trials where only *Sesarma* were placed in the mesocosm. Species-specific, *Carcinus* size-specific and non-consumptive mechanism trials were analyzed with one-way ANOVAs with escape time as the response variable. Data was pooled by treatment for analysis with Bonferroni corrections used to calculate experiment-wide error for avoidance response trials (α’ = 0.017).

We also tested species- and size-specific predation in field mesocosms. Species were placed within flat bottom circular (radius 9 cm) or rectangular mesocosms (42.5 x 30.2 cm) depending on trial species size, that were filled with fresh seawater, covered with hardware cloth mesh to prevent crabs from escaping, and staked into the marsh overnight. All trials included a *Sesarma* (1.95±0.03 cm carapace width), and either had no predatory crab, a large *Carcinus* (6.2±0.1 cm carapace width), small *Carcinus* (3.7±0.2 cm carapace width), *Libinia* (4.1±0.3 cm carapace width), *Cancer* (10.1±0.4 cm carapace width), *Callinectes* (13.0±0.3 cm carapace width), or *Limulus* (18.7±1.2 cm carapace width). Predation events were scored the following morning. Species-specific and *Carcinus* size-specific predation rates were analyzed with one-way ANOVAs with *Sesarma* mortality as the response variable.

## Results

### Why do *Carcinus* use *Sesarma* burrows?

**Figure 1:**
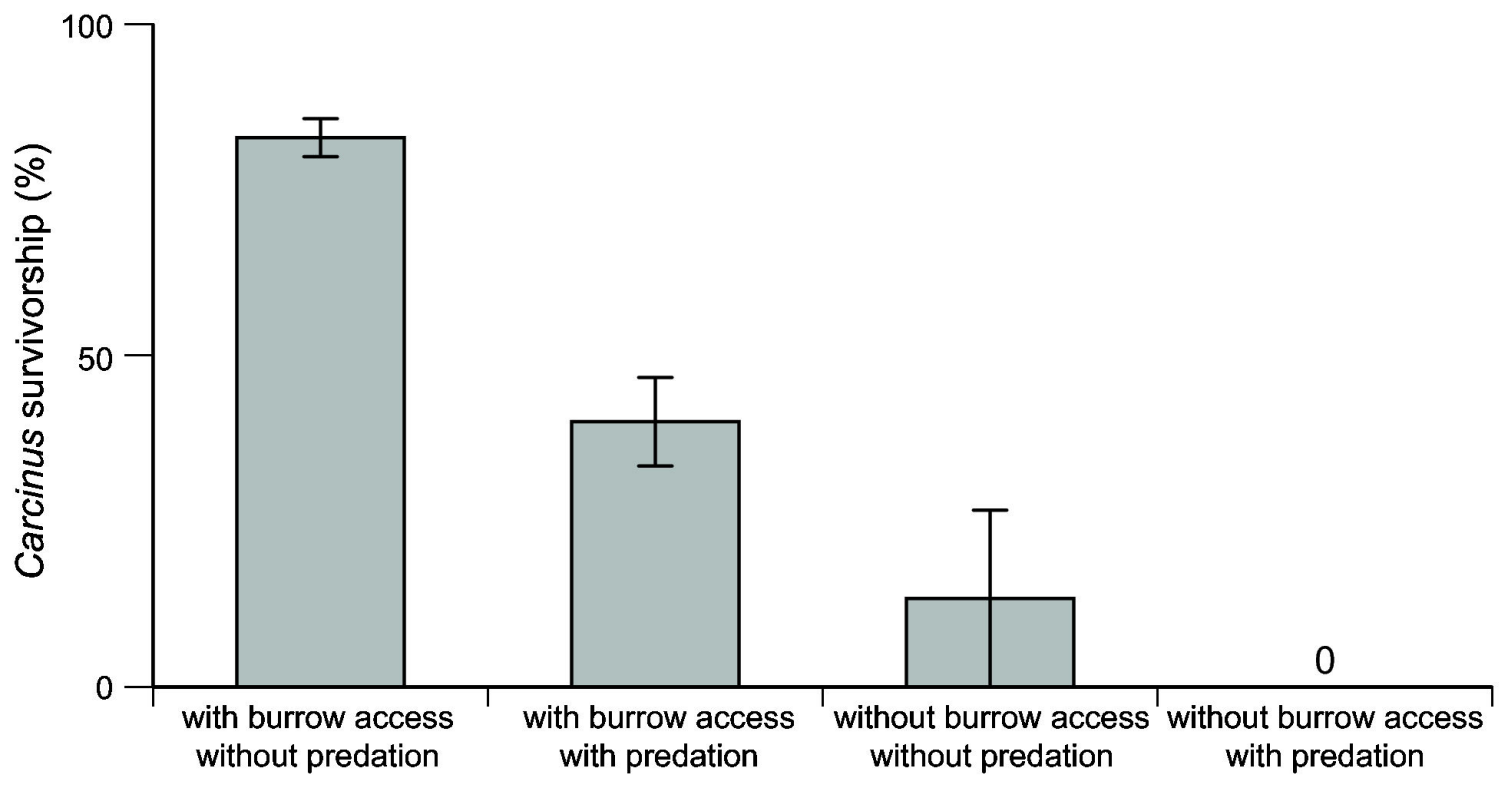
Differential *Carcinus* survivorship with and without burrows access and predation exposure. Tethered *Carcinus* with access to burrows to avoid desiccation and in cages to avoid predation experienced the highest survivorship, while those exposed to both stressors experienced significant mortality. These results underscore the role of *Sesarma* burrows as refuges from desiccation, which transform inhospitable die-off banks into benign intertidal habitats capable of sustaining large, burrow-dwelling *Carcinus* populations.

*Carcinus* had higher mortality when exposed to predation and/or restricted from burrows (predation effect, *F*_1,4_ = 13.70, *P* < 0.05; burrow effect, *F*_1,4_ = 52.07, *P* < 0.01; Figure 1). By restricting burrow access, exposure to physical stress alone led to higher mortality than exposure to predation, but both treatments resulted in higher mortality rates than the predator exclusion with burrow access treatment. All *Carcinus* that lacked burrow access had clear signs of desiccation mortality, while mortality events in replicates without cages left broken, predated carapaces. *Carcinus* exposed to predation without burrows experienced the highest mortality, but the interaction between exposure to predation and burrow access was not significant (*F*_1,4_ = 3.78, *P* = 0.12, Figure 1).

**Figure 2:**
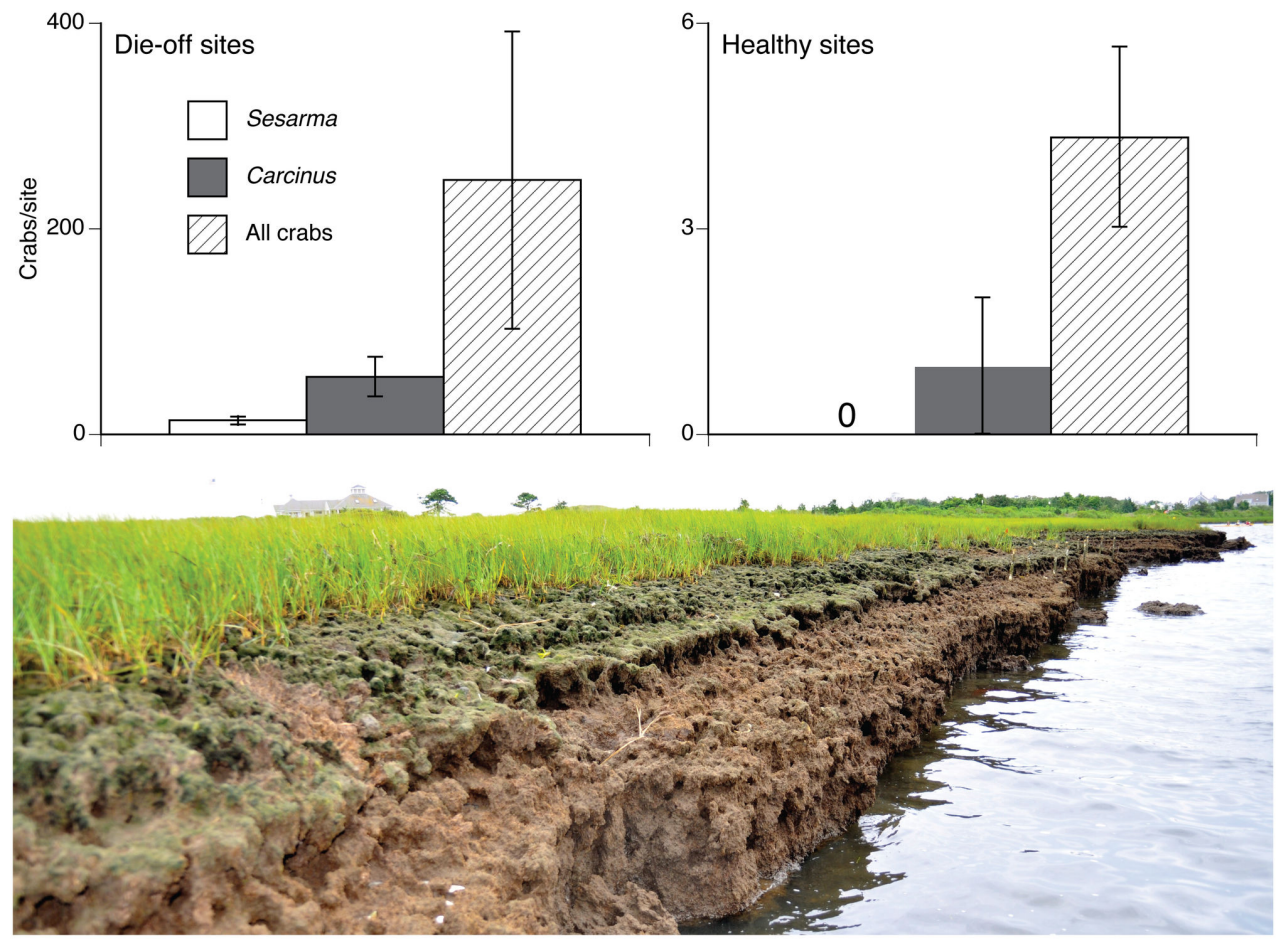
Abundance of intertidal *Sesarma* and *Carcinus* in creek banks at healthy and die-off sites. Note the order of magnitude difference in crab densities between site types. *Carcinus* outnumbered *Sesarma* at both sites, but were only common at sites with high *Sesarma* densities and consequently many burrow complexes and expansive die-off (bottom). *Carcinus* and *Sesarma* were never found in the same burrow and no evidence of predation was ever observed, suggesting *Sesarma* may exhibit a strong avoidance response to the presence of *Carcinus*.

**Figure 3:**
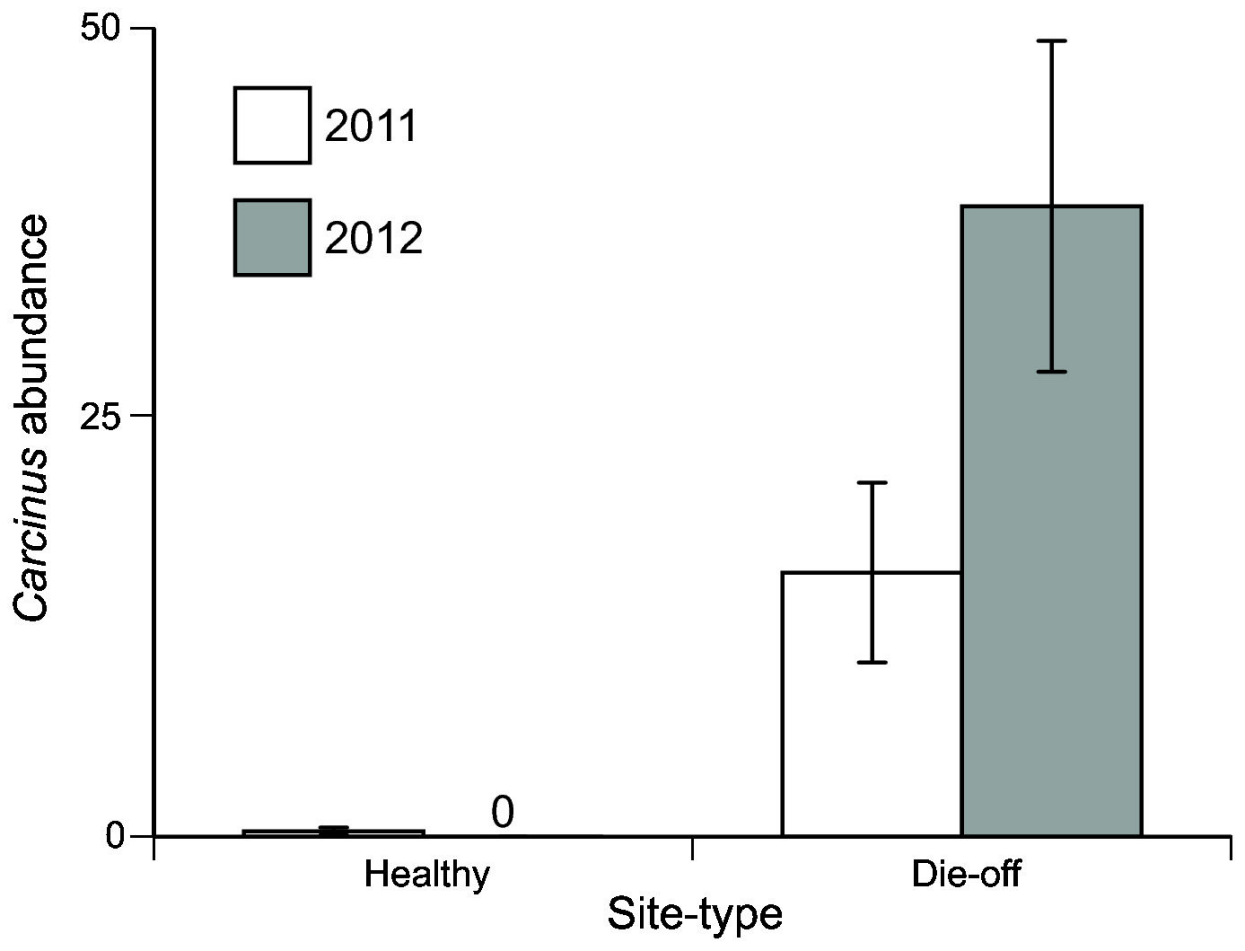
Abundance of intertidal *Carcinus* per creek banks at healthy and die-off sites between 2011 and 2012. Note not only the magnitude difference in *Carcinus* densities between site types but also the difference in abundance trends across years. At healthy sites, *Carcinus* remains low over both 2011 and 2012. At die-off sites, however, *Carcinus* increases from 2011 to 2012.

Recovering marshes have higher burrow densities and wider burrow complexes than healthy sites [36] and we found that *Carcinus* were >50X more common at burrowed, die-off sites than healthy sites with few *Sesarma* burrows (*F*_1,4_ = 7.73, *P* < 0.05). *Sesarma* density was also higher at die-off sites (*F*_1,4_ = 13.23, *P* = 0.02), but *Sesarma* and *Carcinus* were never found in the same burrow (Figure 2). Over two years, *Carcinus* abundance was again higher at die-off than healthy sites (F_1,1_ = 90.678, *P* < 0.05) and increased between 2011 and 2012 (F_1,1_ = 4.54, *P* < 0.05). There was also an interaction between site-type and year (*F*_1,1_ = 5.89, *P* < 0.05). *Carcinus* abundance increased from 2011 to 2012 at die-off sites but remained zero at healthy sites (Figure 3).

### Do *Carcinus* reduce *Sesarma* functional density and herbivory?

**Figure 4:**
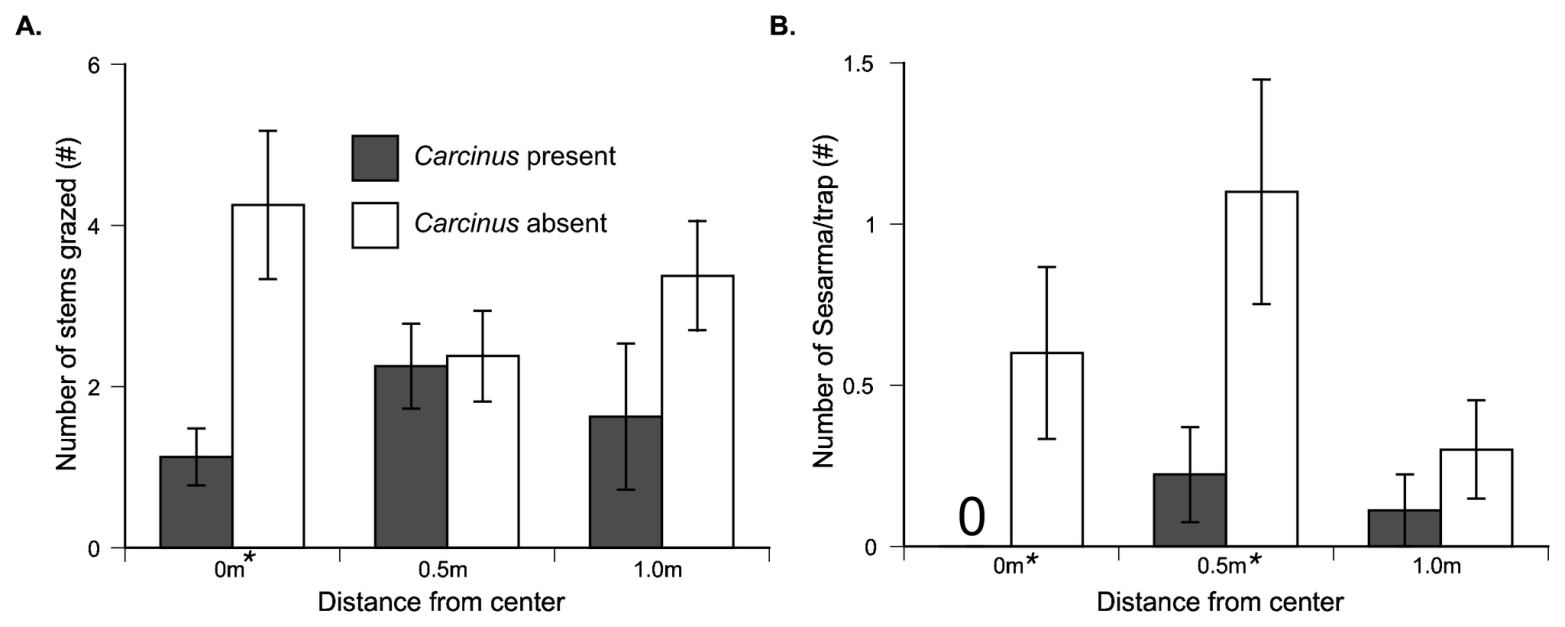
*Carcinus* addition reduces *Sesarma* functional density and herbivory across a spatial gradient. (A) *Sesarma* grazing was reduced by the presence of a single, tethered *Carcinus* at 0m, and (B) *Sesarma* density was reduced at 0 and 0.5m but there was no evidence of predation, which is commonly seen in healthy marshes. This suggests that a single, large *Carcinus* can reduce *Sesarma* functional density and herbivory without directly consuming *Sesarma* (* denotes significant difference at *P* < 0.05).

Before *Carcinus* addition, functional *Sesarma* densities were similar at all distances in all plots (all *F*_1,42_ < 4.25, all *P* > 0.05). Forty eight hours after *Carcinus* addition, *Sesarma* functional density decreased in pitfall traps 0 m (F_1,42_ = 4.53, *P* < 0.05) and 0.5 m (F_1,42_ = 4.98, *P* < 0.05) from the tethered *Carcinus* with >3X decrease in *Sesarma* density at all distances (Figure 4B). *Carcinus* addition reduced *Sesarma* grazing over the duration of the experiment, an effect that decreased with distance (0 m: *F*_1,41_ = 10.06, *P* < 0.0068; 0.5 m: *F*_1,42_ = 0.03, *P* = 0.87; 1.0 m: *F*_1,42_ = 2.39, *P* = 0.35; Figure 4A) and was significant only in the center of experimental plots. There was no evidence of predation on *Sesarma* during the course of the experiment.

### What cues trigger an avoidance response by *Sesarma*?

**Figure 5:**
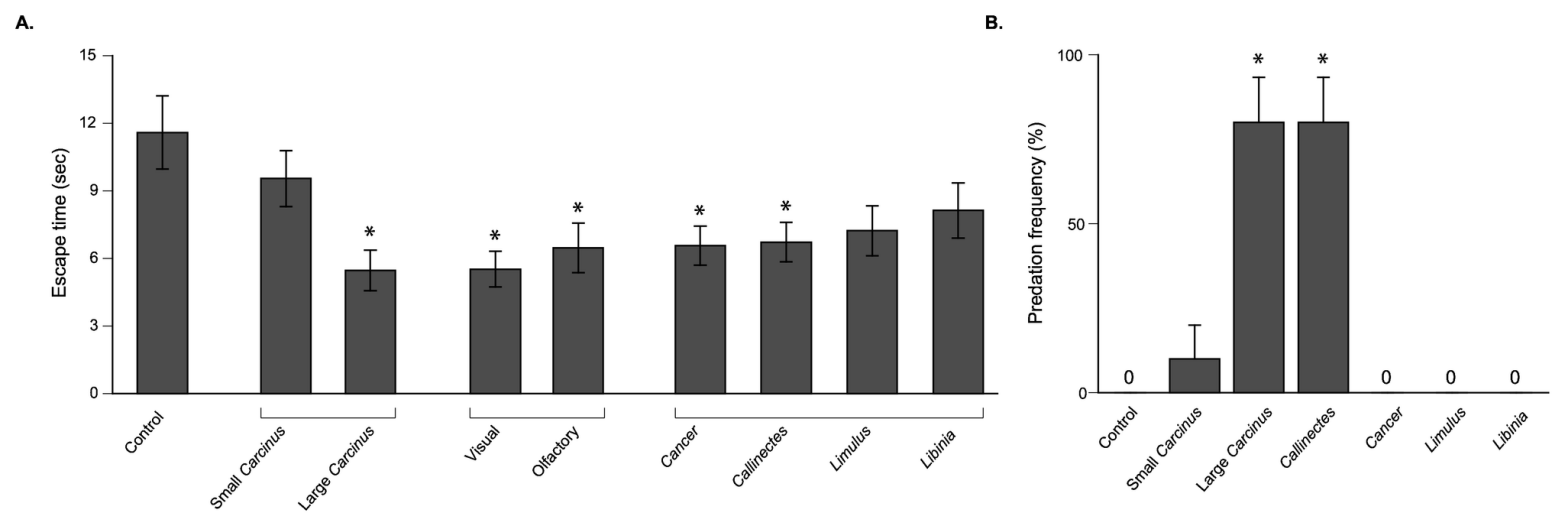
*Sesarma* exhibit species- and size-specific induced avoidance responses reflective of differences in predation rates. (A) Escape response trials demonstrated that *Sesarma* flee faster in response to large *Carcinus*, *Carcinus* visual and olfactory cues, and other similarly sized predatory decapod crustaceans. Non-predatory crustaceans, small *Carcinus*, and *Sesarma* only trials were similar in the amount of time taken to exit the arena. (B) Large *Carcinus* and *Callinectes* preyed on *Sesarma* in overnight feeding trials, but predation was low for small *Carcinus*, *Cancer, Libinia*, and *Limulus* (* denotes significant difference at *P* < 0.05).

*Carcinus* and *Callinectes* were the only species to prey on *Sesarma* in feeding trials (Figure 5B); there was no *Sesarma* mortality in *Sesarma* only trials or in trials with *Libinia, Cancer, or* *Limulus* . *Carcinus* predation on *Sesarma* was size-specific (F_2,27_ = 20.52, *P* < 0.0001), with higher predation rates by large (6.2±0.1 cm) than by small *Carcinus* (3.7±0.2 cm). *Sesarma* also exhibited size-specific avoidance to *Carcinus* (*F*_2,132_ = 8.37, *P* = 0.0004), with large *Carcinus* eliciting an avoidance response ~2X faster than small *Carcinus* and *Sesarma*-only controls (Figure 5A). All *Sesarma* left the arena after ~11 seconds, mimicking the rapid movement of foraging *Sesarma* observed in nearby die-off patches. Olfactory and visual cues elicited similar escape responses (*F*_2,134_ = 7.93, *P* = 0.0006; Figure 5A). Avoidance responses were not limited to *Carcinus*: *Sesarma* avoided all predatory crabs (*Carcinus*, *Callinectes* and *Cancer*; F_5,269_ =3.54, *P* = 0.0041, Figure 5A), but common non-predatory crabs ( *Limulus* and *Libinia*) did not elicit an avoidance response.

## Discussion

Our results suggest that *Carcinus* colonize the intertidal at die-off marshes by using *Sesarma* burrows as refuges from predation and desiccation. At these sites, our results support earlier experiments suggesting that *Carcinus* displace *Sesarma*, exposing them to increased thermal stress and predation [33,36]. This displacement increases *Sesarma* vulnerability to native predators and reduces foraging activity through consumptive and non-consumptive effects, facilitating cordgrass recovery. These results highlight the potential for invasive species to play positive roles outside of their native range [8], particularly when critical ecological functions have been lost due to human impacts.

### Carcinus use of *Sesarma* burrows

Our data suggest that *Carcinus* opportunistically invade the intertidal zone of salt marshes on Cape Cod with high *Sesarma* densities and depleted predator populations and, in the absence of native predators, are becoming numerically dominant predators at die-off marshes [32]. *Carcinus* are unable to burrow in peat and are reliant on large *Sesarma* burrow complexes to invade marsh creek banks (Figure 2). Experimental tethering illustrated that *Carcinus* survival is significantly higher when given access to burrows (Figure 1), suggesting that *Sesarma* burrows provide *Carcinus* a refuge from predation and desiccation at low tide. Mud crabs ( *Panopeus* *herbstii* ) and Asian shore crabs ( *Hemigrapsus* *sanguineus* ) were also found in intertidal creek banks and were similar in size to *Sesarma*. *Carcinus* has been shown to be a superior competitor over *Hemigrapsus* [38] and laboratory feeding trials using *Panopeus* and *Hemigrapsus* (Bertness, unpublished data) have shown no evidence of predation on *Sesarma*, suggesting that *Panopeus* and *Hemigrapsus* likely have no impact on the interaction between *Carcinus* and *Sesarma*. *Panopeus* burrows are too small to be invaded by *Carcinus* and *Hemigrapsus* is not a burrowing crab, further suggesting these species have no effect on *Carcinus* and its dependence on *Sesarma* burrows for persistence. As a result, *Carcinus* reliance on *Sesarma* burrows likely explains the high density of *Carcinus* in the intertidal zone of marshes with severe die-off and high densities of *Sesarma* and their relative absence from sites without burrows (Figure 2).

### *Carcinus* influence on *Sesarma* functional density and cordgrass regrowth

Historically, *Sesarma* densities were controlled by native marine predators such as striped bass ( *Morone* *saxatilis* ), blue crabs ( *Callinectes* *sapidus* ), and smooth dogfish ( *Mustelus* *canis* ). However, decades of recreational fishing have depleted local predator populations within New England salt marshes, releasing *Sesarma* from top-down control. *Carcinus* invasion at die-off sites, however, is partially restoring the predation pressure lost to recreational fishing. By inhabiting *Sesarma* burrow complexes, *Carcinus* effects on *Sesarma* are likely greater *per capita* than those of native predators which are unable to forage both in- and outside of intertidal burrows during low tide.

Our data also suggests that *Carcinus* reduce *Sesarma* activity through visual and olfactory cues (Figure 5A). The magnitude of *Sesarma* response to visual and olfactory cues was similar, and when presented with both stimuli simultaneously, their response was not amplified. These results, coupled with the generic response to predatory crabs exhibited in escape trials with *Callinectes* and *Cancer*, suggest that *Sesarma* are sensitive to visual and olfactory cues from *Carcinus* despite its relatively recent invasion of the Western Atlantic [39].

Our temporal data also illustrates that *Carcinus* have remained at low densities at healthy sites and, coincident with recovery, have been increasing at die-off marshes (Figure 3). Therefore, the recent regrowth of cordgrass into formerly denuded creek banks harboring burrow-dwelling *Carcinus* [36,40] suggests that *Carcinus* is playing a role in promoting the recovery of salt marshes from die-off through both consumptive and non-consumptive effects. Our *Carcinus* addition experiment (Figure 4) revealed that a single *Carcinus* is capable of reducing *Sesarma* activity and increasing the growth and survivorship of nearby cordgrass. While this effect is limited to <1 m, with *Carcinus* densities approaching 10 crabs/m^3^at heavily invaded sites, the consumptive and non-consumptive effects of *Carcinus* burrow invasion are likely strong enough to drive marsh-wide regrowth. While consumptive effects may be playing a role in the marsh recovery, we have observed few naturally predated *Sesarma* body parts in the intertidal at die-off sites (Coverdale, personal observation), and none were found in our *Carcinus* tethering experiment, suggesting that non-consumptive effects may be more prevalent. Similar non-consumptive effects have been shown to produce strong, cascading effects on rocky shores [17], freshwater lakes [41] and terrestrial grasslands [42]. By invading burrow complexes, evicting resident *Sesarma* [36], and living within *Sesarma* burrows, *Carcinus* may also indirectly reduce *Sesarma* densities by enhancing the effectiveness of depleted native predators.

The restriction of the recent *Carcinus* colonization of intertidal creek banks to heavily burrowed marshes suggests that *Sesarma* burrowing facilitates compensatory predation by *Carcinus*, potentially creating a negative feedback loop whereby elevated *Sesarma* densities create conditions suitable for predator colonization. By creating a novel intertidal habitat with refuge from predation and desiccation, *Sesarma* burrows facilitate *Carcinus* invasion into the intertidal zone of predator-depleted marshes, where *Carcinus* suppress *Sesarma* activity and herbivory, promoting cordgrass regrowth and facilitating the recovery of die-off marshes [36,40]. In the absence of burrows at healthy sites, *Carcinus* are vulnerable to desiccation in the intertidal, suggesting that intertidal *Carcinus* and *Sesarma* population fluctuations may be linked in the future. Our results illustrate the severity of human impacts in this system by suggesting that consumptive and non-consumptive top-down control, mediated by an invasive predator, may be facilitating the recovery of heavily degraded Cape Cod salt marshes.
